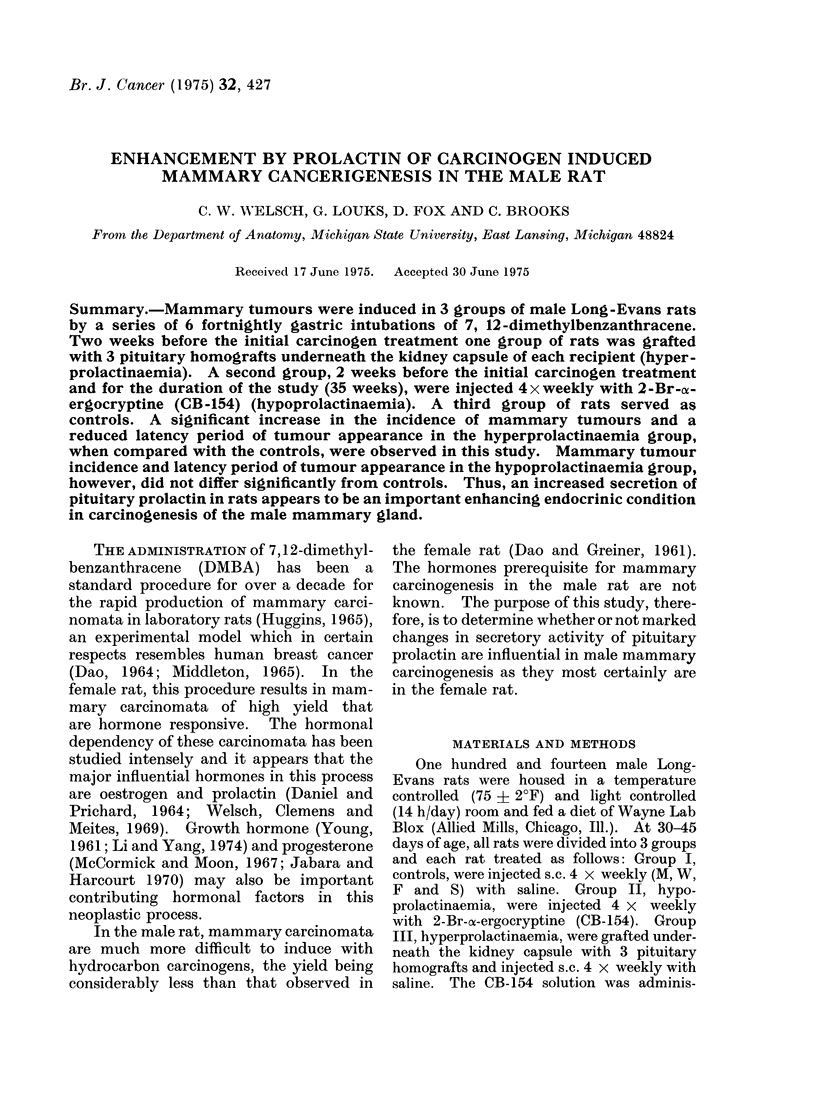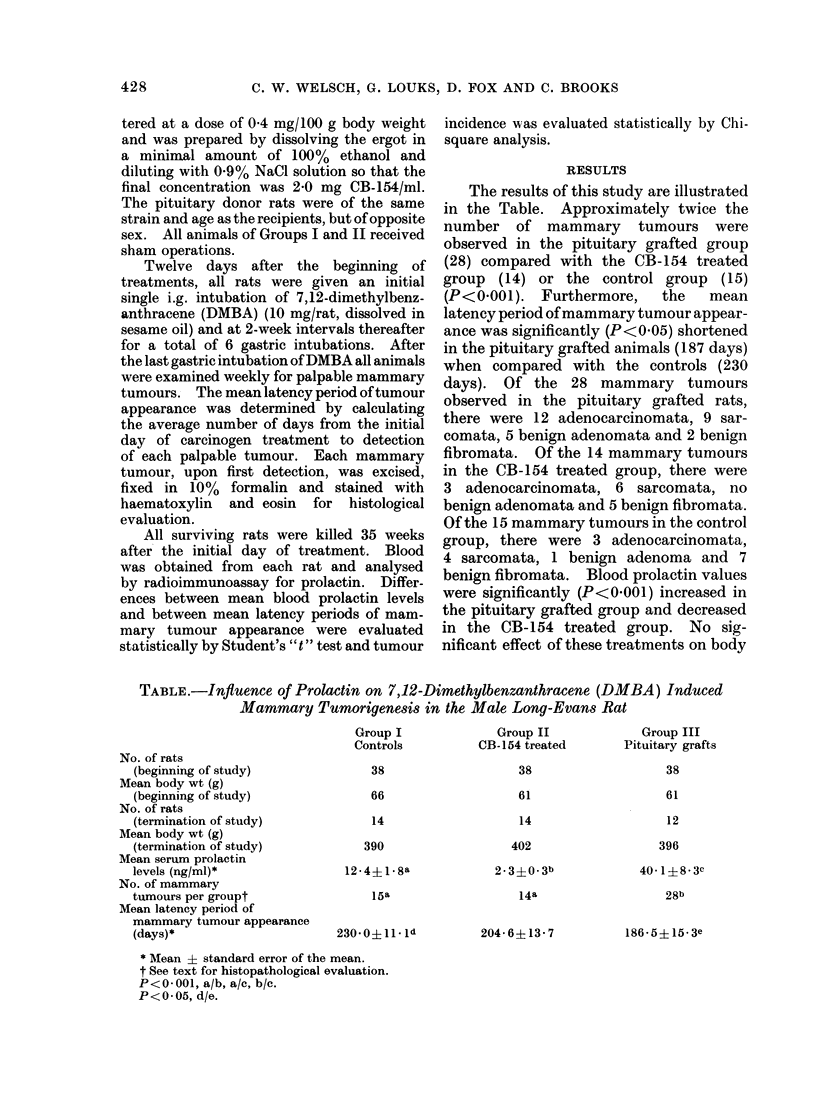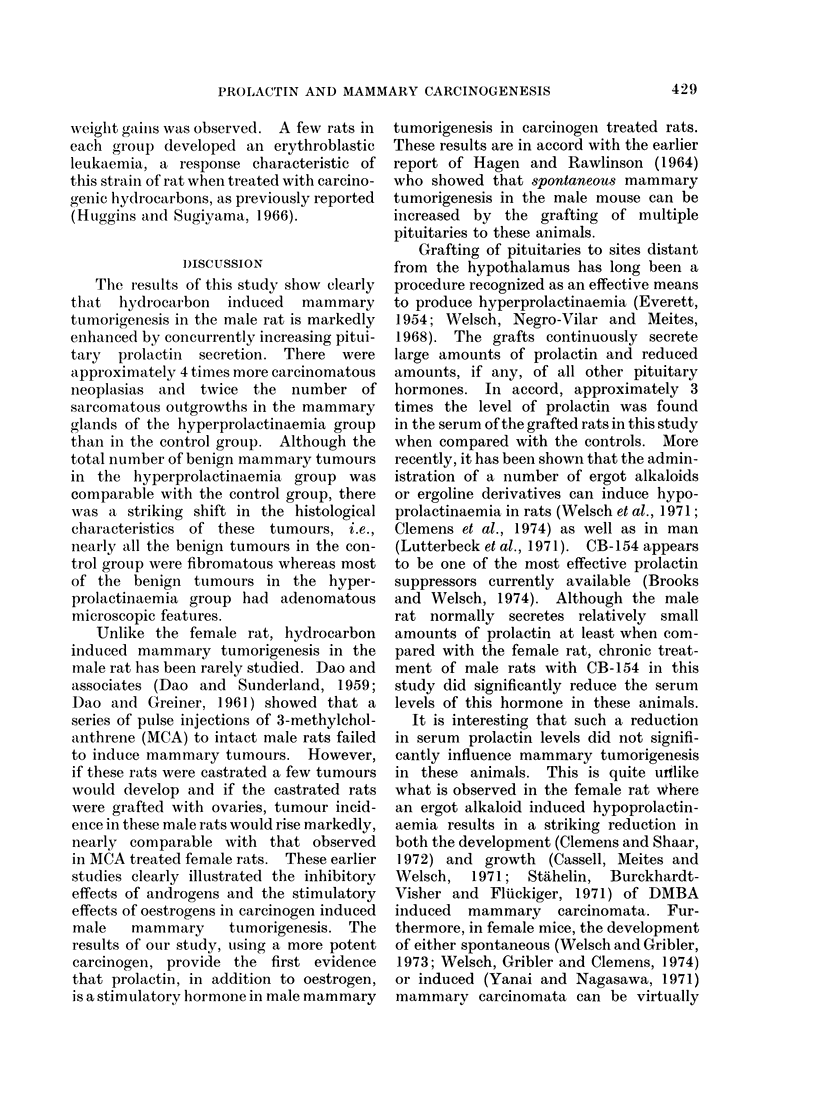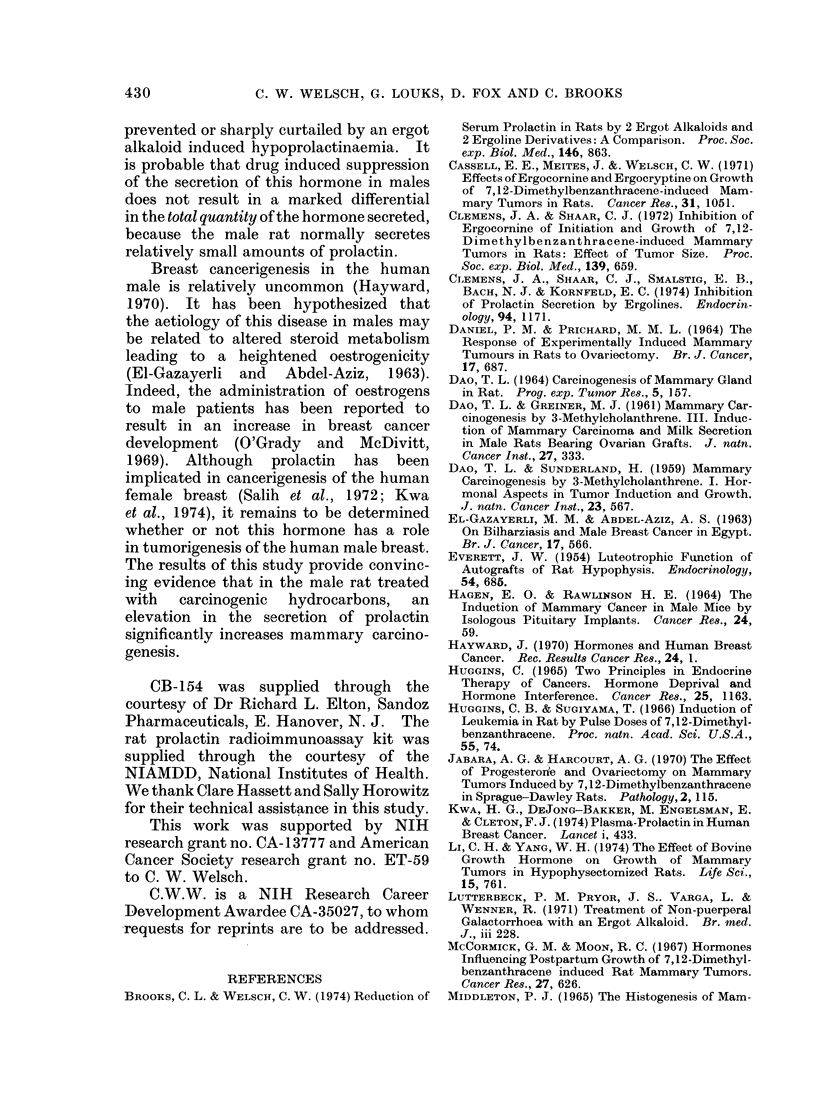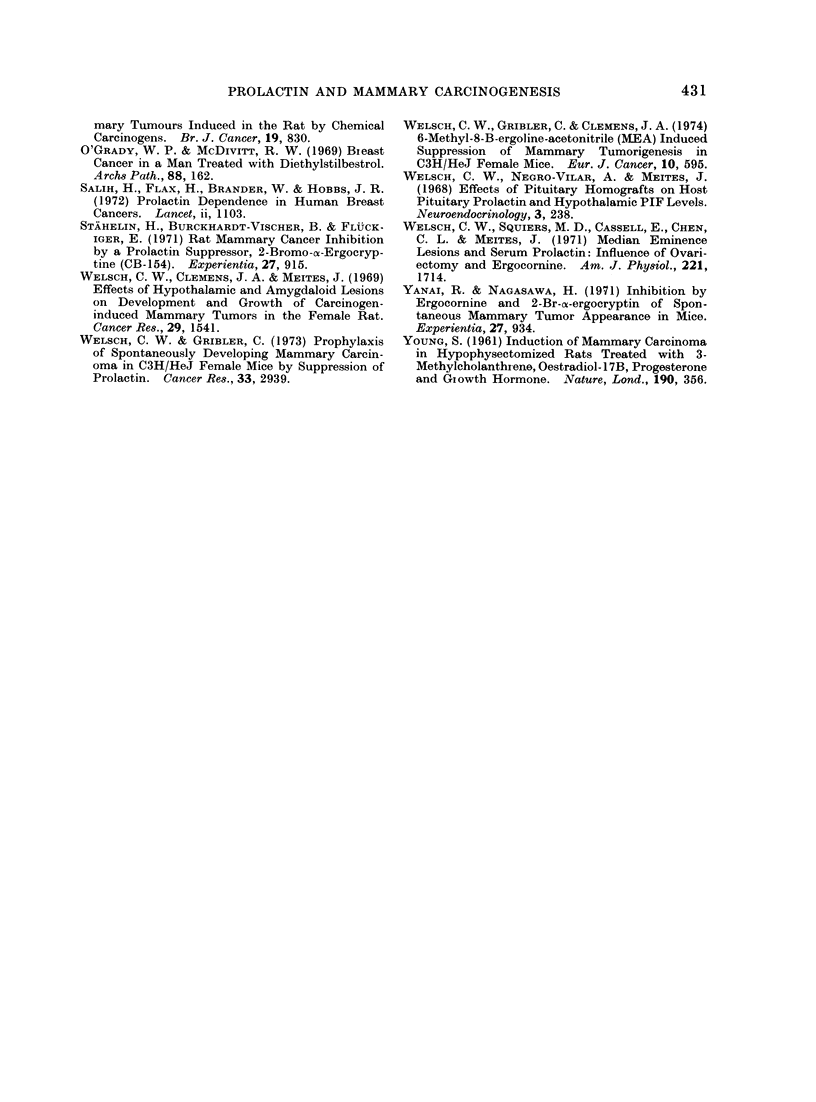# Enhancement by prolactin of carcinogen induced mammary cancerigenesis in the male rat.

**DOI:** 10.1038/bjc.1975.243

**Published:** 1975-10

**Authors:** C. W. Welsch, G. Louks, D. Fox, C. Brooks

## Abstract

Mammary tumours were induced in 3 groups of male Long-Evans rats by a series of 6 fortnightly gastric intubations of 7,12-dimethylbenzanthracene. Two weeks before the initial carcinogen treatment one group of rats was grafted with 3 pituitary homografts underneath the kidney capsule of each recipient (hyperprolactinaemia). A second group, 2 weeks before the initial carcinogen treatment and for the duration of the study (35 weeks), were injected 4 X weekly with 2-Br-alpha-ergocryptine (CB-154) (hypoprolactinaemia). A third group of rats served as controls. A significant increase in the incidence of mammary tumours and a reduced latency period of tumour appearance in the hyperprolactinaemia group, when compared with the controls, were observed in this study. Mammary tumour incidence and latency period of tumour appearance in the hypoprolactinaemia group, however, did not differ significantly from controls. Thus, an increased secretion of pituitary prolactin in rats appears to be an important enhancing endocrinic condition in carcinogenesis of the male mammary gland.


*Br. J. Cancer* (1975) **32**, 427

### ENHANCEMENT BY PROLACTIN OF CARCINOGEN INDUCED MAMMARY CANCERIGENESIS IN THE MALE RAT

C. W. WELSCH, G. LOUKS, D. FOX AND C. BROOKS

*From the Department of Anatomy, Michigan State University, East Lansing, Michigan* 48824

Received 17 June 1975. Accepted 30 June 1975

**Summary.—Mammary tumours were induced in 3 groups of male Long-Evans rats by a series of 6 fortnightly gastric intubations of 7, 12-dimethylbenzanthracene. Two weeks before the initial carcinogen treatment one group of rats was grafted with 3 pituitary homografts underneath the kidney capsule of each recipient (hyperprolactinaemia). A second group, 2 weeks before the initial carcinogen treatment and for the duration of the study (35 weeks), were injected 4 × weekly with 2-Br-α-ergocryptine (CB-154) (hypoprolactinaemia). A third group of rats served as controls. A significant increase in the incidence of mammary tumours and a reduced latency period of tumour appearance in the hyperprolactinaemia group, when compared with the controls, were observed in this study. Mammary tumour incidence and latency period of tumour appearance in the hypoprolactinaemia group, however, did not differ significantly from controls. Thus, an increased secretion of pituitary prolactin in rats appears to be an important enhancing endocrinic condition in carcinogenesis of the male mammary gland.**

THE ADMINISTRATION of 7,12-dimethylbenzanthracene (DMBA) has been a standard procedure for over a decade for the rapid production of mammary carcinomata in laboratory rats (Huggins, 1965), an experimental model which in certain respects resembles human breast cancer (Dao, 1964; Middleton, 1965). In the female rat, this procedure results in mammary carcinomata of high yield that are hormone responsive. The hormonal dependency of these carcinomata has been studied intensely and it appears that the major influential hormones in this process are oestrogen and prolactin (Daniel and Prichard, 1964; Welsch, Clemens and Meites, 1969). Growth hormone (Young, 1961; Li and Yang, 1974) and progesterone (McCormick and Moon, 1967; Jabara and Harcourt 1970) may also be important contributing hormonal factors in this neoplastic process.

In the male rat, mammary carcinomata are much more difficult to induce with hydrocarbon carcinogens, the yield being considerably less than that observed in the female rat (Dao and Greiner, 1961). The hormones prerequisite for mammary carcinogenesis in the male rat are not known. The purpose of this study, therefore, is to determine whether or not marked changes in secretory activity of pituitary prolactin are influential in male mammary carcinogenesis as they most certainly are in the female rat.

### MATERIALS AND METHODS

One hundred and fourteen male Long-Evans rats were housed in a temperature controlled (75 ± 2°F) and light controlled (14 h/day) room and fed a diet of Wayne Lab Blox (Allied Mills, Chicago, Ill.). At 30–45 days of age, all rats were divided into 3 groups and each rat treated as follows: Group I, controls, were injected s.c. 4 × weekly (M, W, F and S) with saline. Group II, hypoprolactinaemia, were injected 4 × weekly with 2-Br-α-ergocryptine (CB-154). Group III, hyperprolactinaemia, were grafted underneath the kidney capsule with 3 pituitary homografts and injected s.c. 4 × weekly with saline. The CB-154 solution was administered at a dose of 0·4 mg/100 g body weight and was prepared by dissolving the ergot in a minimal amount of 100% ethanol and diluting with 0·9% NaCl solution so that the final concentration was 2·0 mg CB-154/ml. The pituitary donor rats were of the same strain and age as the recipients, but of opposite sex. All animals of Groups I and II received sham operations.

Twelve days after the beginning of treatments, all rats were given an initial single i.g. intubation of 7,12-dimethylbenz-anthracene (DMBA) (10 mg/rat, dissolved in sesame oil) and at 2-week intervals thereafter for a total of 6 gastric intubations. After the last gastric intubation of DMBA all animals were examined weekly for palpable mammary tumours. The mean latency period of tumour appearance was determined by calculating the average number of days from the initial day of carcinogen treatment to detection of each palpable tumour. Each mammary tumour, upon first detection, was excised, fixed in 10% formalin and stained with haematoxylin and eosin for histological evaluation.

All surviving rats were killed 35 weeks after the initial day of treatment. Blood was obtained from each rat and analysed by radioimmunoassay for prolactin. Differences between mean blood prolactin levels and between mean latency periods of mammary tumour appearance were evaluated statistically by Student's "*t*" test and tumour incidence was evaluated statistically by Chi-square analysis.

### RESULTS

The results of this study are illustrated in the Table. Approximately twice the number of mammary tumours were observed in the pituitary grafted group (28) compared with the CB-154 treated group (14) or the control group (15) ($P<0{\cdot}001$). Furthermore, the mean latency period of mammary tumour appearance was significantly ($P<0{\cdot}05$) shortened in the pituitary grafted animals (187 days) when compared with the controls (230 days). Of the 28 mammary tumours observed in the pituitary grafted rats, there were 12 adenocarcinomata, 9 sarcomata, 5 benign adenomata and 2 benign fibromata. Of the 14 mammary tumours in the CB-154 treated group, there were 3 adenocarcinomata, 6 sarcomata, no benign adenomata and 5 benign fibromata. Of the 15 mammary tumours in the control group, there were 3 adenocarcinomata, 4 sarcomata, 1 benign adenoma and 7 benign fibromata. Blood prolactin values were significantly ($P<0{\cdot}001$) increased in the pituitary grafted group and decreased in the CB-154 treated group. No significant effect of these treatments on body

TABLE.—*Influence of Prolactin on 7,12-Dimethylbenzanthracene (DMBA) Induced Mammary Tumorigenesis in the Male Long-Evans Rat*

| | Group I Controls | Group II CB-154 treated | Group III Pituitary grafts |
|---|---|---|---|
| No. of rats (beginning of study) | 38 | 38 | 38 |
| Mean body wt (g) (beginning of study) | 66 | 61 | 61 |
| No. of rats (termination of study) | 14 | 14 | 12 |
| Mean body wt (g) (termination of study) | 390 | 402 | 396 |
| Mean serum prolactin levels (ng/ml)* | $12{\cdot}4 \pm 1{\cdot}8^{a}$ | $2{\cdot}3 \pm 0{\cdot}3^{b}$ | $40{\cdot}1 \pm 8{\cdot}3^{c}$ |
| No. of mammary tumours per group† | $15^{a}$ | $14^{a}$ | $28^{b}$ |
| Mean latency period of mammary tumour appearance (days)* | $230{\cdot}0 \pm 11{\cdot}1^{d}$ | $204{\cdot}6 \pm 13{\cdot}7$ | $186{\cdot}5 \pm 15{\cdot}3^{e}$ |

* Mean ± standard error of the mean.
† See text for histopathological evaluation.
$P<0{\cdot}001$, a/b, a/c, b/c.
$P<0{\cdot}05$, d/e.

weight gains was observed. A few rats in each group developed an erythroblastic leukaemia, a response characteristic of this strain of rat when treated with carcinogenic hydrocarbons, as previously reported (Huggins and Sugiyama, 1966).

### DISCUSSION

The results of this study show clearly that hydrocarbon induced mammary tumorigenesis in the male rat is markedly enhanced by concurrently increasing pituitary prolactin secretion. There were approximately 4 times more carcinomatous neoplasias and twice the number of sarcomatous outgrowths in the mammary glands of the hyperprolactinaemia group than in the control group. Although the total number of benign mammary tumours in the hyperprolactinaemia group was comparable with the control group, there was a striking shift in the histological characteristics of these tumours, *i.e.*, nearly all the benign tumours in the control group were fibromatous whereas most of the benign tumours in the hyperprolactinaemia group had adenomatous microscopic features.

Unlike the female rat, hydrocarbon induced mammary tumorigenesis in the male rat has been rarely studied. Dao and associates (Dao and Sunderland, 1959; Dao and Greiner, 1961) showed that a series of pulse injections of 3-methylcholanthrene (MCA) to intact male rats failed to induce mammary tumours. However, if these rats were castrated a few tumours would develop and if the castrated rats were grafted with ovaries, tumour incidence in these male rats would rise markedly, nearly comparable with that observed in MCA treated female rats. These earlier studies clearly illustrated the inhibitory effects of androgens and the stimulatory effects of oestrogens in carcinogen induced male mammary tumorigenesis. The results of our study, using a more potent carcinogen, provide the first evidence that prolactin, in addition to oestrogen, is a stimulatory hormone in male mammary tumorigenesis in carcinogen treated rats. These results are in accord with the earlier report of Hagen and Rawlinson (1964) who showed that *spontaneous* mammary tumorigenesis in the male mouse can be increased by the grafting of multiple pituitaries to these animals.

Grafting of pituitaries to sites distant from the hypothalamus has long been a procedure recognized as an effective means to produce hyperprolactinaemia (Everett, 1954; Welsch, Negro-Vilar and Meites, 1968). The grafts continuously secrete large amounts of prolactin and reduced amounts, if any, of all other pituitary hormones. In accord, approximately 3 times the level of prolactin was found in the serum of the grafted rats in this study when compared with the controls. More recently, it has been shown that the administration of a number of ergot alkaloids or ergoline derivatives can induce hypoprolactinaemia in rats (Welsch *et al.*, 1971; Clemens *et al.*, 1974) as well as in man (Lutterbeck *et al.*, 1971). CB-154 appears to be one of the most effective prolactin suppressors currently available (Brooks and Welsch, 1974). Although the male rat normally secretes relatively small amounts of prolactin at least when compared with the female rat, chronic treatment of male rats with CB-154 in this study did significantly reduce the serum levels of this hormone in these animals.

It is interesting that such a reduction in serum prolactin levels did not significantly influence mammary tumorigenesis in these animals. This is quite unlike what is observed in the female rat where an ergot alkaloid induced hypoprolactinaemia results in a striking reduction in both the development (Clemens and Shaar, 1972) and growth (Cassell, Meites and Welsch, 1971; Stähelin, Burckhardt-Visher and Flückiger, 1971) of DMBA induced mammary carcinomata. Furthermore, in female mice, the development of either spontaneous (Welsch and Gribler, 1973; Welsch, Gribler and Clemens, 1974) or induced (Yanai and Nagasawa, 1971) mammary carcinomata can be virtually prevented or sharply curtailed by an ergot alkaloid induced hypoprolactinaemia. It is probable that drug induced suppression of the secretion of this hormone in males does not result in a marked differential in the *total quantity* of the hormone secreted, because the male rat normally secretes relatively small amounts of prolactin.

Breast cancerigenesis in the human male is relatively uncommon (Hayward, 1970). It has been hypothesized that the aetiology of this disease in males may be related to altered steroid metabolism leading to a heightened oestrogenicity (El-Gazayerli and Abdel-Aziz, 1963). Indeed, the administration of oestrogens to male patients has been reported to result in an increase in breast cancer development (O'Grady and McDivitt, 1969). Although prolactin has been implicated in cancerigenesis of the human female breast (Salih *et al.*, 1972; Kwa *et al.*, 1974), it remains to be determined whether or not this hormone has a role in tumorigenesis of the human male breast. The results of this study provide convincing evidence that in the male rat treated with carcinogenic hydrocarbons, an elevation in the secretion of prolactin significantly increases mammary carcinogenesis.

CB-154 was supplied through the courtesy of Dr Richard L. Elton, Sandoz Pharmaceuticals, E. Hanover, N. J. The rat prolactin radioimmunoassay kit was supplied through the courtesy of the NIAMDD, National Institutes of Health. We thank Clare Hassett and Sally Horowitz for their technical assistance in this study.

This work was supported by NIH research grant no. CA-13777 and American Cancer Society research grant no. ET-59 to C. W. Welsch.

C.W.W. is a NIH Research Career Development Awardee CA-35027, to whom requests for reprints are to be addressed.

### REFERENCES

Brooks, C. L. & Welsch, C. W. (1974) Reduction of Serum Prolactin in Rats by 2 Ergot Alkaloids and 2 Ergoline Derivatives: A Comparison. *Proc. Soc. exp. Biol. Med.*, **146**, 863.

Cassell, E. E., Meites, J. &. Welsch, C. W. (1971) Effects of Ergocornine and Ergocryptine on Growth of 7,12-Dimethylbenzanthracene-induced Mammary Tumors in Rats. *Cancer Res.*, **31**, 1051.

Clemens, J. A. & Shaar, C. J. (1972) Inhibition of Ergocornine of Initiation and Growth of 7,12-Dimethylbenzanthracene-induced Mammary Tumors in Rats: Effect of Tumor Size. *Proc. Soc. exp. Biol. Med.*, **139**, 659.

Clemens, J. A., Shaar, C. J., Smalstig, E. B., Bach, N. J. & Kornfeld, E. C. (1974) Inhibition of Prolactin Secretion by Ergolines. *Endocrinology*, **94**, 1171.

Daniel, P. M. & Prichard, M. M. L. (1964) The Response of Experimentally Induced Mammary Tumours in Rats to Ovariectomy. *Br. J. Cancer*, **17**, 687.

Dao, T. L. (1964) Carcinogenesis of Mammary Gland in Rat. *Prog. exp. Tumor Res.*, **5**, 157.

Dao, T. L. & Greiner, M. J. (1961) Mammary Carcinogenesis by 3-Methylcholanthrene. III. Induction of Mammary Carcinoma and Milk Secretion in Male Rats Bearing Ovarian Grafts. *J. natn. Cancer Inst.*, **27**, 333.

Dao, T. L. & Sunderland, H. (1959) Mammary Carcinogenesis by 3-Methylcholanthrene. I. Hormonal Aspects in Tumor Induction and Growth. *J. natn. Cancer Inst.*, **23**, 567.

El-Gazayerli, M. M. & Abdel-Aziz, A. S. (1963) On Bilharziasis and Male Breast Cancer in Egypt. *Br. J. Cancer*, **17**, 566.

Everett, J. W. (1954) Luteotrophic Function of Autografts of Rat Hypophysis. *Endocrinology*, **54**, 685.

Hagen, E. O. & Rawlinson H. E. (1964) The Induction of Mammary Cancer in Male Mice by Isologous Pituitary Implants. *Cancer Res.*, **24**, 59.

Hayward, J. (1970) Hormones and Human Breast Cancer. *Rec. Results Cancer Res.*, **24**, 1.

Huggins, C. (1965) Two Principles in Endocrine Therapy of Cancers. Hormone Deprival and Hormone Interference. *Cancer Res.*, **25**, 1163.

Huggins, C. B. & Sugiyama, T. (1966) Induction of Leukemia in Rat by Pulse Doses of 7,12-Dimethylbenzanthracene. *Proc. natn. Acad. Sci. U.S.A.*, **55**, 74.

Jabara, A. G. & Harcourt, A. G. (1970) The Effect of Progesterone and Ovariectomy on Mammary Tumors Induced by 7,12-Dimethylbenzanthracene in Sprague–Dawley Rats. *Pathology*, **2**, 115.

Kwa, H. G., DeJong–Bakker, M. Engelsman, E. & Cleton, F. J. (1974) Plasma-Prolactin in Human Breast Cancer. *Lancet* i, 433.

Li, C. H. & Yang, W. H. (1974) The Effect of Bovine Growth Hormone on Growth of Mammary Tumors in Hypophysectomized Rats. *Life Sci.*, **15**, 761.

Lutterbeck, P. M. Pryor, J. S.. Varga, L. & Wenner, R. (1971) Treatment of Non-puerperal Galactorrhoea with an Ergot Alkaloid. *Br. med. J.*, iii 228.

McCormick, G. M. & Moon, R. C. (1967) Hormones Influencing Postpartum Growth of 7,12-Dimethylbenzanthracene induced Rat Mammary Tumors. *Cancer Res.*, **27**, 626.

Middleton, P. J. (1965) The Histogenesis of Mammary Tumours Induced in the Rat by Chemical Carcinogens. *Br. J. Cancer*, **19**, 830.

O'GRADY, W. P. & MCDIVITT, R. W. (1969) Bieast Cancer in a Man Treated with Diethylstilbestrol. *Archs Path.*, **88**, 162.

SALIH, H., FLAX, H., BRANDER, W. & HOBBS, J. R. (1972) Prolactin Dependence in Human Breast Cancers. *Lancet*, ii, 1103.

STÄHELIN, H., BURCKHARDT-VISCHER, B. & FLÜCKIGER, E. (1971) Rat Mammary Cancer Inhibition by a Prolactin Suppressor, 2-Bromo-α-Ergocryptine (CB-154). *Experientia*, **27**, 915.

WELSCH, C. W., CLEMENS, J. A. & MEITES, J. (1969) Effects of Hypothalamic and Amygdaloid Lesions on Development and Growth of Carcinogen-induced Mammary Tumors in the Female Rat. *Cancer Res.*, **29**, 1541.

WELSCH, C. W. & GRIBLER, C. (1973) Prophylaxis of Spontaneously Developing Mammary Carcinoma in C3H/HeJ Female Mice by Suppression of Prolactin. *Cancer Res.*, **33**, 2939.

WELSCH, C. W., GRIBLER, C. & CLEMENS, J. A. (1974) 6-Methyl-8-B-ergoline-acetonitrile (MEA) Induced Suppression of Mammary Tumorigenesis in C3H/HeJ Female Mice. *Eur. J. Cancer*, **10**, 595.

WELSCH, C. W., NEGRO-VILAR, A. & MEITES, J. (1968) Effects of Pituitary Homografts on Host Pituitary Prolactin and Hypothalamic PIF Levels. *Neuroendocrinology*, **3**, 238.

WELSCH, C. W., SQUIERS, M. D., CASSELL, E., CHEN, C. L. & MEITES, J. (1971) Median Eminence Lesions and Serum Prolactin: Influence of Ovariectomy and Ergocornine. *Am. J. Physiol.*, **221**, 1714.

YANAI, R. & NAGASAWA, H. (1971) Inhibition by Ergocornine and 2-Br-α-ergocryptin of Spontaneous Mammary Tumor Appearance in Mice. *Experientia*, **27**, 934.

YOUNG, S. (1961) Induction of Mammary Carcinoma in Hypophysectomized Rats Treated with 3-Methylcholanthrene, Oestradiol-17B, Progesterone and Giowth Hormone. *Nature, Lond.*, **190**, 356.